# Antibacterial activity and structural properties of gelatin-based sol-gel synthesized Cu-doped ZnO nanoparticles; promising material for biomedical applications

**DOI:** 10.1016/j.heliyon.2024.e37022

**Published:** 2024-08-28

**Authors:** Nadia Mahmoudi Khatir, Ali Khorsand Zak

**Affiliations:** a Department of Biotechnology, Faculty of Biological Sciences, Alzahra University, Tehran, 1993891176, Iran; b Nanobiotechnology Laboratory, Esfarayen University of Technology, Esfarayen, 96619-98195, North Khorasan, Iran

**Keywords:** ZnO, Lattice strain, Crystallite size, X-ray diffraction, Antibacterial

## Abstract

This study investigates the antibacterial activity and spectral characteristics of Cu-doped ZnO nanoparticles synthesized via the gelatin-based sol-gel method, focusing on their potential biomedical applications. Zn₁₋ₓCuₓO nanoparticles (x = 0.0, 0.01, 0.03, and 0.05) were fabricated using this method. The incorporation of copper dopants into the ZnO matrix significantly influences both the crystalline structure and spectral properties of the nanoparticles. X-ray diffraction analysis confirms the presence of a wurtzite structure without any pyrochlore phase. The broadening of spectral features indicates modifications in lattice parameters and elastic constants. XRD results reveal that adding Cu to the ZnO lattice causes a decrease in crystallite size from 32 to 18 nm. Transmission electron microscopy shows spherical-shaped ZnO nanoparticles with sizes ranging from 30 to 40 nm. Moreover, Cu-doped ZnO nanoparticles exhibit considerable inhibition against bacterial growth. Adding Cu enhances the antibacterial activity of ZnO nanoparticles, suggesting their potential in biomedical applications. Overall, these findings highlight the promising prospects of sol-gel synthesized Cu-doped ZnO nanoparticles in the biomedical field.

## 1 Introduction

Zinc oxide (ZnO) is known as metal oxide semiconductor (n-type) with a wide band gap of 3.32 eV. It is highly valued for its unique properties, including high exciton-binding energy (60 meV), a large velocity of saturation about 3.2 × 10^7^ cm/s, and its non-toxicity [1,2]. Typically, ZnO has a hexagonal structure with a c/a ratio of 1.65, although this can vary based on growth conditions [3]. Over the years, various synthesis methods have been developed to control the growth of ZnO nanostructures, including sol-gel [4,5], hydrothermal [6], solvothermal [7], sonochemical [8], combustion [9], chemical vapor deposition (CVD) [10], vapor-liquid-solid (VLS) [11], laser ablation [12], precipitations [13], and pulsed laser deposition (PLD) [14].

The crystallite size effects, particularly quantum confinement, significantly influence the ZnO particles properties, especially at the nanometric scale where defects become more prominent [15]. Doping ZnO with other elements is known a promising approach to tuning its properties [16]. The lattice parameters and crystallite size are substantially affected by the ionic radii difference of the dopants and zinc [[17], [18], [19]]. Therefore, it is crucial to consider these phenomena in crystallite calculations. Lattice strain and crystallite size are the main factors contributing to the broadening of diffraction peaks. Lattice strain, caused by lattice imperfections or dopants, alters the peak intensity and position. The peak position is shifted by uniform strain, while non-uniform strain increases peak broadening [3]. Recently, there have been renewed concern in developing antibacterial covering for infection debarment. For example, reactive oxygen species metal oxides those using non-selective microbicides are remarkable materials for this propose [20]. Based on the literature, synthesis of pure ZnO in various morphologies and its antibacterial and antimicrobial properties have been extensively investigated [21,22]. Proposed mechanisms for this activity include the production of reactive oxygen species, hydrogen peroxide, superoxide anions (O^2−^), and hydroxyl radicals generations [23,24], the extrication of Zn^2+^ ions [25], damage of cell membrane [26], and the accumulations of nanoparticles on the outer membranes and in the cytoplasm [27]. However, the antibacterial and antimicrobial properties of Cu-doped ZnO nanoparticles are not yet fully understood, requiring precise synthesis methods and systematic characterizations [[28], [29], [30]]. Cu-doped ZnO is studied for antibacterial purposes due to several compelling reasons including.

- The combination of ZnO and Cu can create a synergistic effect, leading to enhanced antibacterial properties compared to pure ZnO or Cu alone.
- Copper ions (Cu^2^⁺) are known to disrupt bacterial cell membranes, generate reactive oxygen species (ROS), and interfere with enzyme functions, thereby effectively killing bacteria.
- ZnO nanoparticles are known to generate ROS under UV or visible light irradiation. Doping with Cu can enhance this ROS generation, which in turn damages bacterial cells by oxidative stress.
- Doping ZnO with Cu can alter its electronic and structural properties, such as bandgap energy, which can enhance its photocatalytic and antibacterial efficiency.
- Cu doping can improve the stability of ZnO nanoparticles and lead to more uniform particle morphology, which is beneficial for consistent antibacterial performance.
- Both ZnO and Cu have broad-spectrum antibacterial activity, meaning they can be effective against a wide range of bacterial strains, including both Gram-positive and Gram-negative bacteria.
- ZnO is generally recognized as safe (GRAS) and is already used in various biomedical applications. When doped with Cu, the resulting nanoparticles can maintain low toxicity and high biocompatibility, making them suitable for biomedical applications.
- Beyond antibacterial properties, Cu-doped ZnO nanoparticles can also have potential applications in other areas such as photocatalysis, sensor technology, and drug delivery, making them versatile and valuable in various fields.

These factors make Cu-doped ZnO nanoparticles a promising material for antibacterial studies and potential biomedical applications. Cu-doped ZnO nanoparticles have been synthesized using various methods to study their antibacterial behavior [[31], [32], [33]]. For example, Raju et al. prepared Cu-doped ZnO nanoparticles using the co-precipitation method. However, this method has certain drawbacks, such as residual impurities like agents or polymers remaining after washing, which can affect the results. Additionally, the morphology of the products is often not uniform [34]. Therefore, the synthesis method used for Cu-doped nanoparticles plays a crucial role and significantly affects the properties of the final products.

Using the gelatin-based sol-gel method, we prepare un-doped and Cu-doped ZnO nanoparticles, with gelatin serving as the polymerization agent to control nanoparticle growth. By using this method, very fine and uniform powders are obtained. Also, this method is very simple and low cost. In addition, Cu atoms are well-dispersed in the ZnO lattice structure. We investigate the variation of copper concentration on the optical and structural properties of the prepared ZnO nanoparticles and determine their antibacterial property.

## 2 Materials and methods

### 2.1 Materials

Analytical grade zinc nitrate hexahydrate (Zn(NO_3_)_2_·6H_2_O), copper nitrate hexahydrate (Cu(NO_3_)_2_·6H_2_O), gelatin ((NHCOCH–R1)n, R1 = amino acid, Type A, Porcine), and distilled water (DW) are used as starting raw materials for the synthesis of nanoparticles.

### 2.2 Nanoparticles synthesis

To prepare 2 g of the un-doped and doped ZnO product first specific amounts of copper and zinc nitrates are dissolved in 25 ml of DW. The nitrate amounts are calculated based on the Zn_1–x_Cu_x_O formula, with x values of 0, 0.01, 0.03, and 0.05 as the fallowing equation (1).

(1) $$\left(1-x\right)\text{Zn}{\left({\text{NO}}_{3}\right)}_{2}∙6H_{2}O+\text{xCu}{\left({\text{NO}}_{3}\right)}_{2}∙6H_{2}O\to {Zn}_{1-x}{Cu}_{x}O+\cdots$$

4 g of gelatin are slowly added to 70 ml of DW (maintaining a gelatin to the final product ratio of 2:1) at 70 °C. Once the gelatin is fully dissolved, a clear homogenous solution is obtained. Then, the Cu^2+^ and Zn^2+^ solution is poured into the gelatin solution. To control the temperature and fix it at 85 °C, the mixed solution is stirred in an oil bath. The thermal treatment is continued for 6 h and stirred until a viscous gel forms. To perform the calcination step, the inner wall of the crucible is smeared with some of the obtained gel before placing it in the furnace. The furnace operates at a fixed temperature of 600 °C for 2 h. The heating rate is applied as 5 °C/min and then it remains to rich room temperature naturally.

### 2.3 Characterizations

The crystalline structures of the un-doped and Cu-doped ZnO Nanoparticles are confirmed via X-ray diffraction (XRD) measurements using a D5000 diffractometer with Cu-Kα1 radiation (1.54056 Å) at 40 kV and 100 mA. The scanning range of angle (2θ) is from 0 to 40°, employing a slow scanning speed of approximately 1.2°/min. (resolution: 0.011). The shape, size, and morphology of the Nanoparticles are observed using transmission electron microscopy (TEM, Hitachi H-7100) and scanning electron microscopy (SEM, FESEM, Quanta 200F).

#### 2.3.1 Growth of bacteria and antibacterial tests

The antibacterial activity of the nanoparticles was assessed using the minimum inhibitory concentration (MIC) method, performed by microdilution in a microtiter plate [35]. MIC is the lowest concentration of an antimicrobial compound that inhibits the growth of microorganisms in a dilution sensitivity test. Each well received 100 μl of MHB culture medium, and 100 μl of each nanoparticle solution was added to the first well (each experiment was repeated three times) and then serially diluted (0.5–256 μg/ml). Two standard bacterial strains, *Staphylococcus aureus* ATCC 25922 and *Escherichia coli* ATCC 1431, were inoculated into each well with approximately 5 × 10⁵ CFU/ml cells. The turbidity and bacterial growth in each microplate were determined after 18–24 h of incubation at 37 °C. Controls included culture medium with bacterial inoculum (bacterial control), culture medium with each nanoparticle (nanoparticle control), and MHB culture medium alone (media control). A milky homogeneous suspension appeared after sonication in any of the nanoparticle samples. The antibacterial effect of the nanoparticles and the results of bacterial growth under their influence were determined by the turbidity observed after incubation at 37 °C.

For the antibacterial activity evaluations of the prepared nanoparticles, *E. coli* microorganisms were cultivated following a specific protocol. First, nutrient broth (8 g) was dissolved in 1000 ml of DW and heated until fully dissolved. After that, the solution was autoclaved at 120 °C and pressure of 15 lbs for 15 min to sterilize it. After sterilization, the nutrient broth was incubated at 35 °C for 30 h with *E. coli* (the gram-negative bacterium). The impact of the Nanoparticles on bacterial growth inhibition is assessed using an agar diffusion method. *E. coli* was grown in the nutrient broth, and determining of the optical density at a wavelength of 600 nm for the density measurement of bacterial cells existing in liquid cultures [36].

## 3 Results and discussions

### 3.1 XRD analyses

**Fig. 1:**
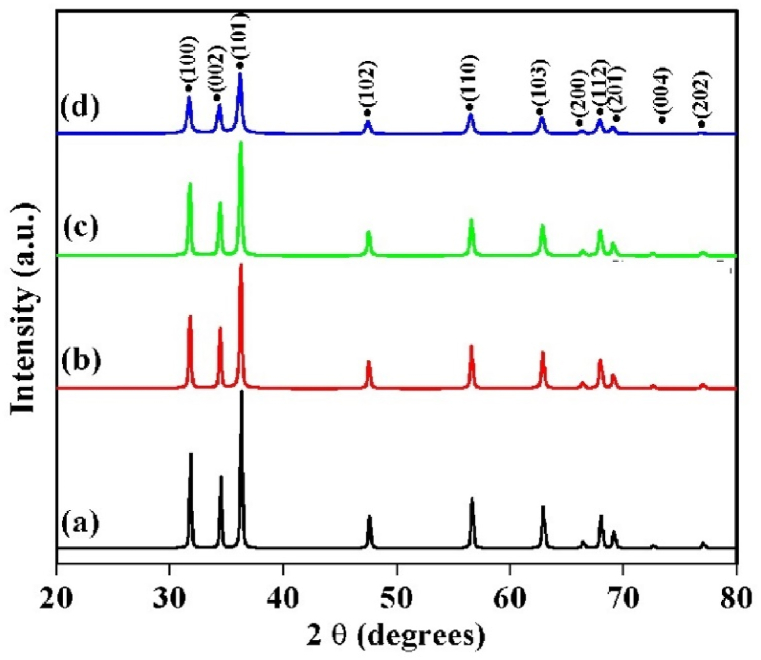
XRD patterns related to the prepared un-doped and Cu-doped ZnO nanoparticles.

Fig. 1 shows the XRD pattern of the prepared un-doped and Cu-doped ZnO nanoparticles. The obtained diffraction peaks are remarked as the hexagonal structure of ZnO, conforming to PDF Code: 00-036-1451. It is seen that, the height of the detected diffraction peaks decreases with copper content increases. This change and decrease in the peak intensity is related to the structural defects or disorders introduced by the copper ions in the ZnO lattice structure. The lattice parameters are presented in Table 1. No additional peaks were observed adjacent to the main peaks, which are attributed to other structures or compounds. This indicates that the introduction of copper ions did not disrupt the hexagonal structure of zinc oxide and effectively replaced zinc ions within the lattice. This outcome aligns with expectations given the proximity of copper to zinc atoms in the periodic table and their similar ionic radii.

**Table 1:** The lattice parameters of the prepared pure and doped samples.

Compound	2 θ± 0.01	hkl	d_hkl_(nm)±0.0005	Structure	Lattice parameter (nm) ± 0.0005	V(Å^3^)±0.0002
ZnO	31.78 34.43	(100) (002)	0.2813 0.2602	Hexagonal	a = 0.3252 c = 0.5204	0.0476
Zn_0.99_Cu_0.01_O	31.82 34.49	(100) (002)	0.2809 0.2598	Hexagonal	a = 0.3247 c = 0.5196	0.0474
Zn_0.97_Cu_0.03_O	31.70 34.39	(100) (002)	0.2819 0.2605	Hexagonal	a = 0.3258 c = 0.5210	0.0478
Zn_0.95_Cu_0.05_O	31.77 34.43	(100) (002)	0.2813 0.2602	Hexagonal	a = 0.3252 c = 0.5204	0.0476

**Fig. 2:**
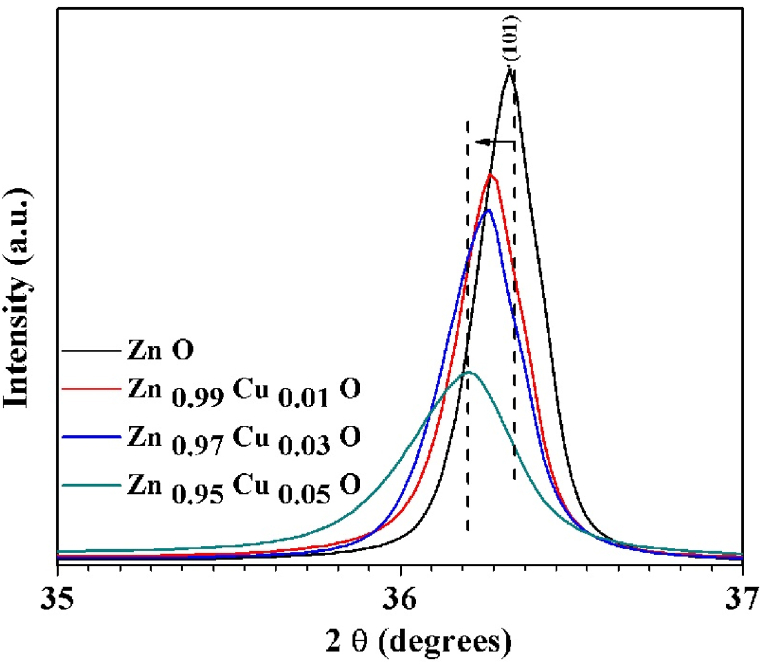
The variation of (101) peak position with increasing Cu.

Fig. 2 clearly indicates a significant shift for the (101) peak position towards a lower angle with increasing copper content in the ZnO matrix. This shift is attributed to the variation of Cu^2^⁺ and Zn^2^⁺ ionic radii, that induces greater lattice strain.

The FWHM evaluations are employed to determine the crystallite size, estimated using Scherrer equation D=Kλβcosθ, where the crystallite size is shown by *D*, wavelength by *λ* (1.54056 Å for Cu-K_α_ radiation), the peak's full width at half-minimum intensity (*FWHM*) by *β, K* is a constant about 0.94, the peak position as *θ*. (101) diffraction peak the maximum intensity is chosen for the calculations. It is important to mention that Scherrer equation has limitations in accurately estimating size because the lattice strains caused by defects or imperfections do not accounted in this method. Therefore, other methods, such as the Williamson-Hall plot, are necessary for accurate size estimation. To separate the strain and crystallite size effects on the peak broadening of the sample, the strain is expressed as $\varepsilon \left(\theta \right)=4\varepsilon_{0}\;\tan\;\theta$, where ε0=Δdd (Bragg plane spacing fractional variations). By plotting sinθλ in respect to ε0cosθλ (the plot obtained in Williamson-Hall), the lattice strain is calculated. The presented method defines the relationship among the lattice strain, crystallite size, and peak broadening as follows, Eq. (1):

(1a) $$\beta_{hkl}\;\cos\;\theta =\left(\frac{k\lambda }{D}\right)+\left(4\varepsilon \;\sin\;\theta \right)$$

It is assumed that the diffracting domains and microstrain contributions are isotropic. The new relation in size-strain plot (SSP) method offers a better evaluation and advantage for isotropic line broadening where gives less weight for data achieved at the higher angles. The relationship among strain, peak broadening, and crystallite size in this method is given by Eq. (2) [37]:

(2) $${\left({d_{hkl}\beta }_{hkl}\;\cos\;\theta \right)}^{2}=\frac{k}{D}\left(d_{hkl}^{2}\beta_{hkl}\;\cos\;\theta \right)+{\left(\frac{\varepsilon }{2}\right)}^{2}$$

**Fig. 3:**
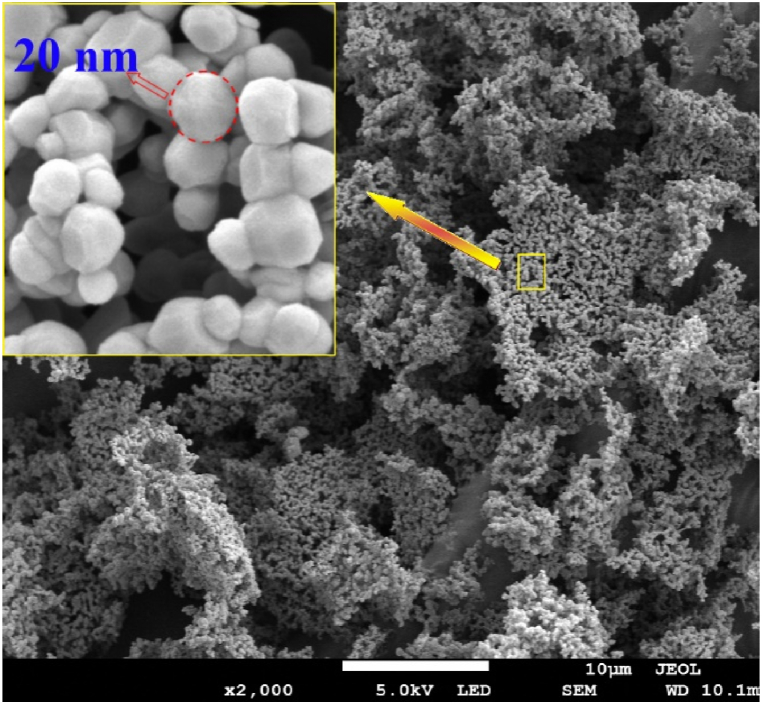
FESEM micrograph of the prepared ZnO nanoparticles.

**Fig. 4:**
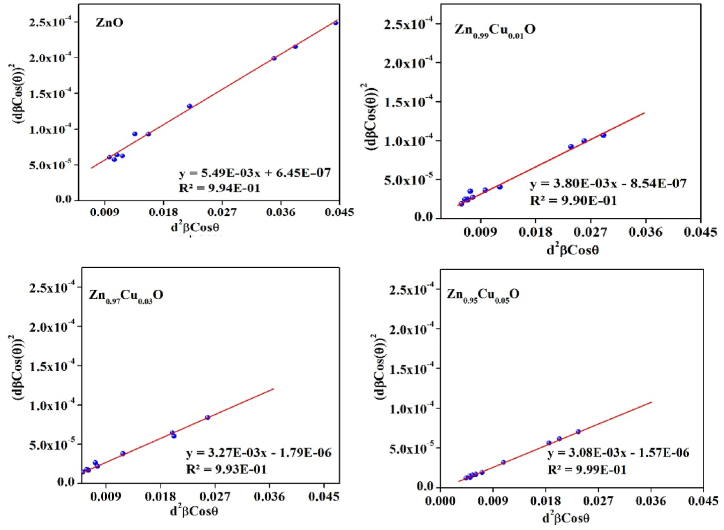
SSP results of the prepared un0-doped and Cu-doped ZnO nanoparticles.

The shape factor k is 3/4 for spherical Nanoparticles. The typical FESEM micrograph shows spherical morphology for the prepared ZnO nanoparticles (Fig. 3). The term ${\left({d_{hkl}\beta }_{hkl}\;\cos\;\theta \right)}^{2}$ is plotted versus $\left(d_{hkl}^{2}\beta_{hkl}\;\cos\;\theta \right)$ and applied for all diffraction peaks appearing between 2 θ = 20° to 80°, and the points are linearly fitted as illustrated in Fig. 4. The slope of the line and its y-intercept give the crystallite size and lattice strain, respectively, the results are listed in Table 1.

Lattice stress and strain has relation according to the Hooke's law, *σ* = *Yε*, (*Y* is the Young's modulus and *σ* is the stress). This linear relationship only responsible for small dislocations and uniform strain. SSP method is chosen to obtain lattice strain and Young's modulus for the hexagonal structure is obtained using the following relationship, Eq. (3) [3]:

(3) $$Y_{hkl}=\frac{{\left[h^{2}+\frac{{\left(h+2k\right)}^{2}}{3}+{\left(\frac{al}{c}\right)}^{2}\right]}^{2}}{s_{11}{\left(h^{2}+\frac{{\left(h+2k\right)}^{2}}{3}\right)}^{2}+s_{33}{\left(\frac{al}{c}\right)}^{4}+\left(2s_{13}+s_{44}\right)\left(h^{2}+\frac{{\left(h+2k\right)}^{2}}{3}\right){\left(\frac{al}{c}\right)}^{2}}$$

where the ZnO elastic compliances are *s*_*11*_*, s*_*13*_*, s*_*33*_ and *s*_*44*_ have the values 7.858 × 10^−12^, −2.206 × 10^−12^, 6.940 × 10^−12^, and 23.57 × 10^−12^ m^2^N^−1^, respectively. The obtained value of *Y* is 125 GPa for the synthesized ZnO Nanoparticles. Also, we can compute the energy density of the lattice from, Eq. (4):

(4) u=σ22Yhkl=ε2Yhkl2

The results calculated and obtained from the SSP and Scherrer calculations are presented in Table 2. The crystallite size results of the prepared Nanoparticles calculated using both Scherrer and SSP methods show decreases in crystallite size value as the Cu dopant increases. The defects are increased by adding Cu to the ZnO lattice due to the difference of the ionic radii. Also, the speed of the crystal growth is affected by the chemical reactivity of the dopant. The chemical reactivity of copper is lower compared to that of zinc, therefore, adding copper as the dopant into the zinc lattice reduces the growth speed of the ZnO crystal and, consequently, the size of the Nanoparticles [38]. There are differences in size estimates between the Scherrer and SSP methods because the SSP method accounts for strain effects on peak broadening, whereas the Scherrer method does not.

**Table 2:** The variations of the elastic constant and crystallite size under different amounts of Cu.

Compound	Scherrer	Size Strain Plot				
	D(nm)	D(nm)	ε× 10^−3^	Y × 10^9^	σ× 10^7^	u × 10^4^
**ZnO**	46	32	1.6	125	20	16
**Zn**_**0.99**_**Cu**_**0.01**_**O**	43	30	1.8	125	22.5	20.25
**Zn**_**0.97**_**Cu**_**0.03**_**O**	36	26	2.7	125	33.75	45.56
**Zn**_**0.95**_**Cu**_**0.05**_**O**	25	18	2.5	125	31.25	39.06

### 3.2 TEM images

**Fig. 5:**
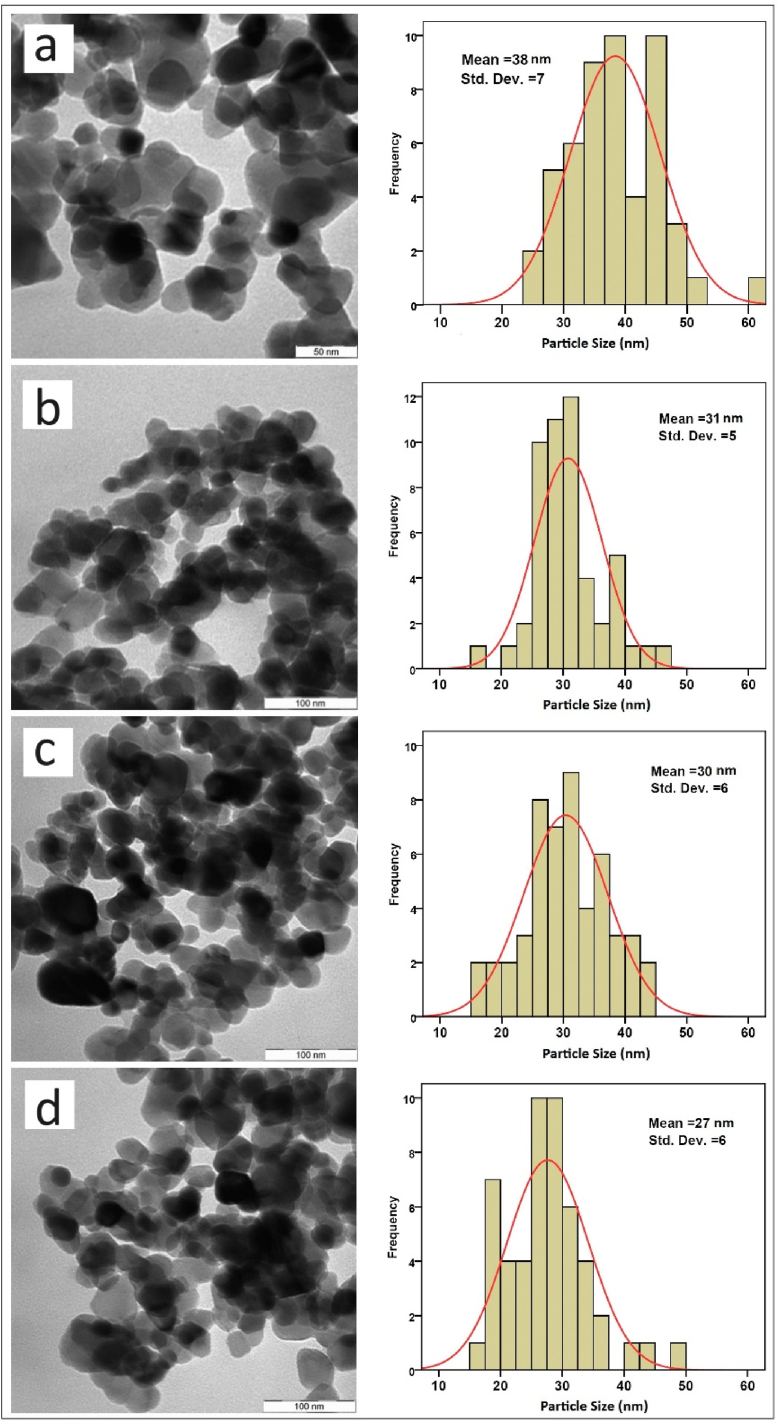
TEM micrograph of the prepared (a) un-doped and (1 %), (3 %), and (5 %) Cu-doped ZnO nanoparticles.

The TEM micrographs for all samples, along with their size distributions, are presented in Fig. 5. The average particle sizes of the Zn_1-x_Cu_x_O (x = 0.0, 0.01, 0.03, and 0.05) nanoparticles are observed to be 38 ± 7 nm, 31 ± 5 nm, 30 ± 6 nm, and 27 ± 6 nm, respectively. Interestingly, the inclusion of Cu in pure ZnO reduces the size of the Nanoparticles compared to the undoped ones. These observations align well with the XRD results, suggesting that the Nanoparticles are nearly single crystals. Additionally, the size distribution is found to be Gaussian.

### 3.3 UV–vis features

**Fig. 6:**
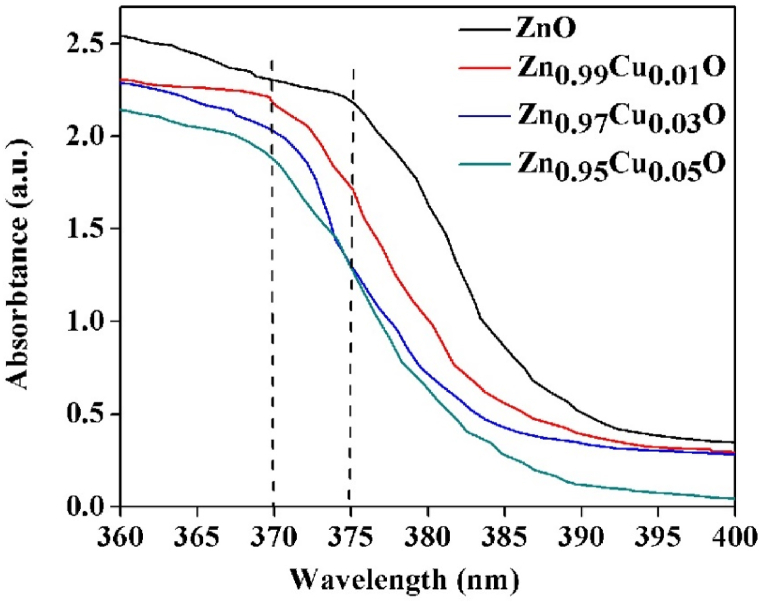
UV–Visible absorption spectra of the prepared pure and Cu-doped ZnO Nanoparticles.

Fig. 6 illustrates the absorption spectra of Cu-doped and undoped ZnO nanoparticles. The blue shift in the absorption peak due to the interpolation of Cu into the ZnO lattice is attributed to defect-mediated transition processes [39]. The creation of localized defect states within the band gap region primarily causes this effect. However, quantum confinement and surface states also play significant roles in the absorption processes. This defect-assisted enhanced absorption may correlate effectively with the antibacterial activities, warranting further careful investigation.

### 3.4 Antibacterial activity

**Fig. 7:**
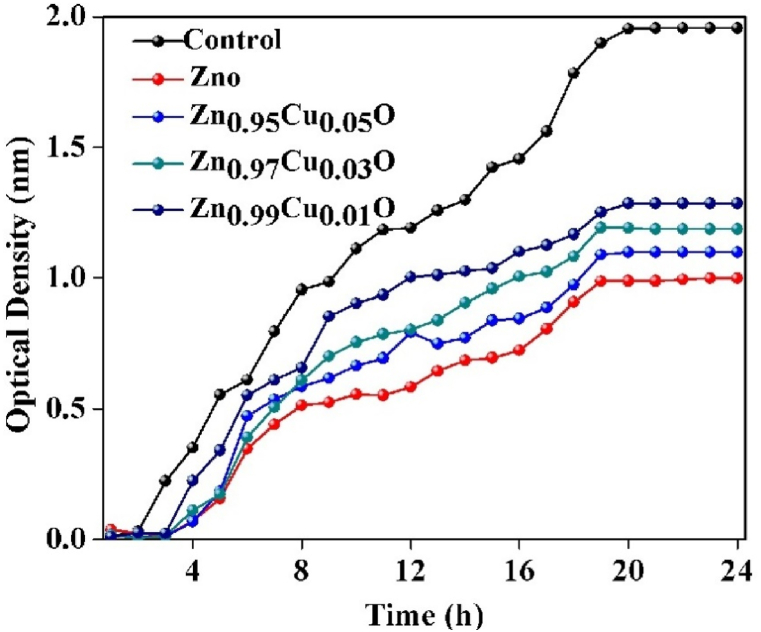
Antibacterial activities of the prepared pure and Cu-doped ZnO Nanoparticles.

Fig. 7 illustrates the antimicrobial potency of ZnO nanoparticles at various Cu concentrations. The antimicrobial activities of Zn_1-x_Cu_x_O Nanoparticles with different Cu levels are tested against *E. coli* that is known as a Gram-negative bacterium. The kinetics of bacterial growth are investigated by using curve of the bacterial inhibition growth to study the antibacterial properties of the prepared doped and un-doped samples. As we expected, the bacterial solution without doped Nanoparticles shows no bacterial growth inhibition. However, significant inhibition occurs at 5 % Cu doping. No noticeable inhibition of *E. coli* is observed at lower Cu dopant concentrations (1 % and 3 %). It is suggested that incorporating Cu into the ZnO matrix can significantly alter the antibacterial activity of the doped Nanoparticles. Nevertheless, Adding copper to zinc oxide reduces the oxidation power of zinc atoms and as a result reduces the antimicrobial effects of zinc.

**Fig. 8:**
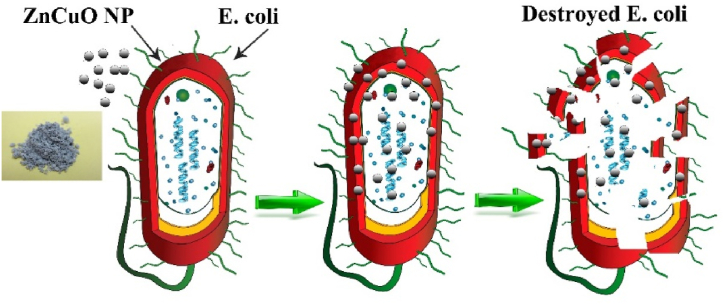
The schematic of interaction of Zn_1-x_Cu_x_O nanoparticles on *E. coli*.

Fig. 8 presents a schematic diagram illustrating the interaction between Zn_1-x_Cu_x_O nanoparticles and an *E. coli* cell with Nanoparticles. At biological pH values, bacteria and spore cells possess an overall negative charge due to an excess of carboxylic and other groups that dissociate, making the cell surface negatively charged [40]. The interaction between these negatively charged bacteria and the released copper ions from the doped Nanoparticles, combined with bioactivity and adhesion, results in the bacterial cell wall destruction and subsequent bacterial death as the copper NP concentration increases [41,42]. The release of Cu^2^⁺ ions escalates with higher NP concentrations, indicating that more free ions are present in the liquid medium, effectively inhibiting growth of bacterial.

To prevent bacterial growth and kill bacteria, a sufficient amount of copper is required in the environment. Studies show that increasing the concentration of copper significantly impacts the growth and mortality of *E. coli*. The extent of this effect is directly proportional to the copper concentration present. Although the exact mechanism and mode of penetration of nanoparticles and their interaction with bacteria are not fully understood, experiments have demonstrated that these nanoparticles alter the morphology of the *E. coli* cell membrane and cause damage to it.

## 4 Conclusions

Spherical-shaped Cu-doped ZnO nanoparticles were synthesized using the gelatin-based sol–gel method. The significant effects of Cu dopants on the structural and antibacterial properties were investigated. Doping Cu into the ZnO lattice affected the crystalline size through strain interactions changed by defect, leading to broadening of the diffraction peaks width. This modification subsequently affected the crystallite size and elastic parameters of the prepared crystalline Nanoparticles’. XRD measurements confirmed the hexagonal wurtzite structure of Zn_1-x_Cu_x_O at this calcination temperatures without any impurity phases. The detected peak broadening was analyzed using the SSP method and Scherrer and the, revealing a decrease in crystallite size with increasing dopant (Cu) concentrations. TEM images showed the presence of ZnO Nanoparticles with a mean particle size ranging from approximately 30 to 40 nm. Furthermore, ZnO Nanoparticles with 5 % Cu dopants exhibited significant inhibition against bacterial growth. These promising results suggest that our synthesized Nanoparticles hold potential for biomedical applications.

## CRediT authorship contribution statement

**Nadia Mahmoudi Khatir:** Writing – original draft, Methodology, Data curation. **Ali Khorsand Zak:** Writing – review & editing, Supervision.

## Declaration of competing interest

The authors declare that they have no known competing financial interests or personal relationships that could have appeared to influence the work reported in this paper.
